# Hydrophobic Collagen/Polyvinyl Alcohol/V_2_CT_x_ Composite Aerogel for Efficient Oil Adsorption

**DOI:** 10.3390/polym17141949

**Published:** 2025-07-16

**Authors:** Erhui Ren, Jiatong Yan, Fan Yang, Hongyan Xiao, Biyu Peng, Ronghui Guo, Mi Zhou

**Affiliations:** 1 College of Biomass Science and Engineering, Sichuan University, Chengdu 610065, China; renerhui0429@163.com (E.R.);; 2 Yibin Industrial Technology Research Institute, Sichuan University, Yibin 644000, China; 3 College of Energy Resources, Sichuan University of Science and Technology, Chengdu 610061, China

**Keywords:** aerogel, absorption, collagen, directional freezing–drying, V_2_CT_x_ MXene

## Abstract

The development of effective oil adsorbents has attracted a great deal of attention due to the increasingly serious problem of oil pollution. A light and porous collagen (COL)/polyvinyl alcohol (PVA)/vanadium carbide (V_2_CT_x_) composite aerogel was synthesized using a simple method of blending, directional freezing, and drying. After modification with methyltriethoxysilane (MTMS) via chemical vapor deposition, the aerogel possessed an excellent hydrophobicity and its water contact angle reached 135°. The hydrophobic COL/PVA/V_2_CT_x_ composite aerogel exhibits a porous structure with a specific surface area of 49 m^2^/g. It also possesses prominent mechanical properties with an 80.5 kPa compressive stress at 70% strain, a low density (about 28 mg/cm^3^), and outstanding thermal stability, demonstrating a 61.02% weight loss from 208 °C to 550 °C. Importantly, the hydrophobic COL/PVA/V_2_CT_x_ aerogel exhibits a higher oil absorption capacity and stability, as well as a faster absorption rate, than the COL/PVA aerogel when tested with various oils. The hydrophobic COL/PVA/V_2_CT_x_ aerogel has the capacity to adsorb 80 times its own weight of methylene chloride, with help from hydrophobic interactions, Van der Waals forces, intermolecular interactions, and capillary action. Compared with the pseudo first-order model, the pseudo second-order model is more suitable for oil adsorption kinetics. Therefore, the hydrophobic COL/PVA/V_2_CT_x_ aerogel can be used as an environmentally friendly and efficient oil adsorbent.

## 1. Introduction

The leakage of oil and organic solvents as a result of production, exploration, use, and transportation processes has recently become the main cause of water pollution [1]. For instance, a collision that occurred in the East China Sea led to a vast oil spill that not only resulted in environmental pollution, but also brought about significant economic losses and damage to ecosystems and health [2]. Considering the increasing spread of oily contaminants, the need to design and prepare a new type of efficient and environmentally friendly oil adsorbent has become urgent [3,4]. Different types of porous organic materials such as polystyrene foam [5] and polyurethane [6], as well as inorganic materials like clay [7] and silica [8], have been employed in the field of oil adsorption. However, these approaches have a low adsorption capacity, are easily filled with oil, and exhibit poor biodegradability, which significantly limits their practical application.

Aerogel, which is a light and porous material, has been widely applied in relation to oil adsorption [9,10]. Among the numerous materials used to synthesize aerogels, biomass containing lignin [11,12], protein, cellulose [13], collagen, and polysaccharide stands out for its environmental impact, ease of acquisition, and low cost [14,15]. Collagen (COL), which is a primary protein, possesses a special structure and rich functional groups like alkyl, aromatic nucleus, amino, and hydroxyl groups, which are necessary for oil removal. Collagen can be found in various extracellular matrices of vertebrates [16,17]. However, the poor mechanical properties of pure collagen aerogels mean that they are crushed in the process of oil adsorption, thus decreasing their adsorption capacity. Some materials, such as polyvinyl alcohol, graphene oxide, sodium carboxymethyl cellulose, and MXene nanosheets, have been added to synthesized collagen-based composite aerogels for tissue engineering [18], synthetic bone graft substitutes [19], dye adsorption, supercapacitors [20], sensitive pressure sensors [21], and other applications [22]. Additionally, a sodium carboxymethyl cellulose/collagen complex aerogel was prepared and applied in relation to dye adsorption, while a wax-coated hydrophobic COL–CMC complex aerogel worked well in relation to oil–water separation [17].

V_2_CT_x_ MXene, which is a type of two-dimensional metallic carbide, is usually prepared by using hydrofluoric acid to wipe off the aluminum (Al) layer that forms during the 3D V_2_AlC phase [23,24]. This V_2_CT_x_, which has an excellent electrical conductivity and contains various functional groups, has been widely used as a compound with reduced graphene oxide (RGO) for applications in battery energy storage, catalytic hydrogen evolution, and dye adsorption [25,26]. V_2_CT_x_ MXene was deposited onto a PVA-coated PVDF through vacuum deposition to prepare a bi-functional membrane that can be applied in the separation of oil and water [27]. The three-dimensional TiVCTX MXene/graphene aerogel was fabricated via freezing–drying; its adsorption capacity toward pump oil reached 42 g/g [28]. The fact that adding V_2_CT_x_ MXene sheets can improve the mechanical properties of composite aerogels has inspired us to introduce V_2_CT_x_ into a COL aerogel. Compared with Ti_3_C_2_T_x_, V_2_CT_x_ has a slightly lower hardness, but possesses an excellent toughness. When it is used for mechanical enhancement, it not only enhances the compressive strength of organic aerogels but also prevents brittle fractures. In addition, directional freeze-drying has been proven to improve the mechanical properties of aerogels [29,30]. Moreover, PVA—which is low cost, biodegradable, and biocompatible [31]—has long chains that form large numbers of hydrogen bonds between COL and V_2_CT_x_, thus enhancing the mechanical properties of the composite aerogels. For instance, CNF/PVA/GO aerogels prepared using directional freeze-drying were found to be excellent oil adsorbents, while highly compressible anisotropic graphene aerogels fabricated through directional freezing were used for adsorbing organic liquids [10].

To promote the oil adsorption capacity of aerogels, it is necessary to carry out hydrophobic modifications [32]. Atomic layer deposition, chemical vapor deposition, and carbonization are commonly used to improve hydrophobicity [4,12,33]. Considering the performance of COL and PVA, methyltriethoxysilane (MTMS) without fluorine or chlorine was selected to improve the hydrophobicity of the aerogel through chemical vapor deposition (CVD). Compared with other methods that require multiple steps of reactions or large amounts of organic solvents, the MTMS modification is more convenient to operate and is more environmentally friendly. The residual methanol can be removed by vacuum drying [34]. Inspired by the advantages of directional freezing–drying and CVD, the COL/PVA/V_2_CT_x_ composite aerogel was prepared with the following raw materials: type Ⅰ collagen, which is stable and easily gained form animal skins; PVA, which is biodegradable, biocompatible, and inexpensive; and V_2_CT_x_, which has a high hardness and excellent stability in this study. The oil adsorbing capacity, mechanical properties, and thermal stability of the hydrophobic COL/PVA/V_2_CT_x_ composite aerogel are significantly improved compared to the hydrophobic COL/PVA aerogel. The V_2_CT_x_ was combined with COL and PVA through oxygen-containing functional groups such as hydroxyl and carboxyl. Meanwhile, the structure of collagen and PVA prevents the oxidization and aggregation of V_2_CT_x_ in the mixed solution. This study prepared a new hydrophobic COL/PVA/V_2_CT_x_ composite aerogel, which exhibits a light weight, a low density, a porous structure, thermal stability, and outstanding mechanical properties; it can be used for efficient oil adsorption.

## 2. Experimental

### 2.1. Materials

V_2_AlC powder (400 mesh), which was putted in a glass dryer, was bought from Forsman Scientific Co., Ltd. (Beijing, China). Type I collagen (from fish; 90%) was stored in the refrigerator and was purchased from MACKLIN reagent (Shanghai, China). Tertbutyl ammonium hydroxide (TBAOH), hydrofluoric acid (HF; 50 wt% solution), and glutaraldehyde (GA; 30%) were provided by Kelong Chemical Co., Ltd. (Chengdu, China). Liquid nitrogen was provided by Xuyuan Chemical Co., Ltd. (Chengdu, China). Methyltrimethoxysilane (MTMS; 99%) was obtained from Titan Scientific Co., Ltd. (Shanghai, China). Kitchen paper was purchased from Vinda Tissues at Walmart supermarket. All materials and chemicals were directly used without any further purification.

### 2.2. Preparation of COL/PVA/V_2_CT_x_ Composite Aerogels

The V_2_CT_x_ MXene used in this work was obtained by etching the V_2_AlC phase with HF, following the methods described in a previous study [35]. In particular, 2 g V_2_AlC powder was slowly added into a 20 mL HF (50 wt%) solution thrice over 10 min, before being sealed in a Teflon bottle and being vigorously stirred at 25 °C for 72 h to remove the aluminum (Al) layer. High concentrations of hydrofluoric acid and the temperature caused excessive etching, while low concentrations of hydrofluoric acid made it hard to wipe off the Al layer. After the obtained reaction mixture was centrifuged, intercalated by TBAOH, and dried in a vacuum oven, the V_2_CT_x_ sheet was successfully prepared.

PVA was dissolved with continuous and vigorous stirring at 80 °C for 2 h to form a 1 wt% concentration solution. Then, a moderate mass of type I collagen was dispersed into the aforementioned PVA solution (which was cooled to room temperature) with stirring and sonication until it became a homogeneous gel-like solution with a 1 wt% concentration. Afterwards, a certain mass of the prepared V_2_CT_x_ sheet was added to the collagen and PVA mixed liquor to obtain the target concentrations of 0 wt%, 0.1 wt%, 0.2 wt%, and 0.3 wt%. Next, the mixed liquids were stirred for 2 min and sonicated for 2 min, in turn; this was repeated three times. Then, 30 uL of glutaraldehyde (GA) was immersed as the cross-linking agent during the stirring process. After the mixture became homogeneous, it was transferred into a polydimethylsiloxane mold and frozen using directional freezing in a homemade device with liquid nitrogen. The homemade device consisted of a 3 L volume Dewar and several copper columns of 2 cm in diameter and 10 cm in height; a portion of the columns was dipped in liquid nitrogen to create a heat sink, where the molds filled with liquid were placed on the end of the bars. The cooling rate was kept at 10 °C/min and the temperature gradient was −196 °C→−56 °C. The frozen samples were lyophilized for 36 h at −50 °C using a freeze-dryer to obtain the COL/PVA and COL/PVA/V_2_CT_x_ composite aerogels.

### 2.3. Development of Hydrophobic Aerogels

The aerogels prepared in Section 2.2 were modified using MTMS via chemical vapor deposition in order to improve the hydrophobicity of the aerogels, which is helpful for oil adsorption. The COL/PVA and COL/PVA/V_2_CT_x_ composite aerogels and a small open glass beaker containing MTMS were all placed in a large sealed container; it should be noted that the aerogels were not directly in contact with the MTMS. Then, the container was heated to 70 °C and was reacted for 4 h at a steam concentration of 0.04 mol/L. After the silanation reaction finished, the aerogel was completely coated with MTMS and became hydrophobic. Excess methyl alcohol that remained in the hydrophobic aerogels was dislodged through vacuum drying at 40 °C until the pressure dropped to below 0.03 mbar. In addition, the fabricated aerogels with different V_2_CT_x_ content ratios (0 wt%, 0.1 wt%, 0.2 wt%, and 0.3 wt%) were appointed as hydrophobic COL/PVA, COL/PVA/V_2_CT_x_—0.1, COL/PVA/V_2_CT_x_—0.2, and COL/PVA/V_2_CT_x_—0.3 composite aerogels, respectively. The density (*ρ*) of the products was calculated according to Equation (1), as follows:

(1) ρ=m/v

where *ρ* is the density of the aerogel, *m* is the mass, and *v* is the volume of the aerogel.

### 2.4. Characterization

The prepared samples including V_2_CT_x_, hydrophobic COL/PVA/V_2_CT_x_, and COL/PVA composite aerogels were investigated using an X-ray diffractometer (XRD) with Cu Kα radiation (λ = 1.54 Å) at 40 kV and 40 mA and a diffraction angle (2θ) transforming from 5° to 70°; Fourier transform infrared spectroscopy (FTIR, Tracer-100, Shimadzu, Japan) in the range of 600–4000 cm^−1^ with a 2 cm^−1^ resolution; and an X-ray photoelectron spectrometer (XPS, Escalab 250Xi, ThermoFisher Scientific, Waltham, MA, USA). The morphologies of the aerogels were characterized with a scanning electron microscopy (SEM, Apero 2c, ThermoFisher Scientific). To analyze the thermal properties of the aerogels, a thermal analyzer (TG209F3, NETZSCH, Selb, Bavaria, Germany) was heated at 10 °C/min from room temperature to 600 °C under N_2_ atmosphere. The compression properties of the aerogels were tested using a universal mechanical testing system in the shape of a cylinder (68TM-30, Instron, Boston, MA, USA) at a rate of 5 mm/min. The water contact angle of the aerogels was assessed using a contact angle goniometer (WCA, HARK100, HARK Beijing, China) in order to determine hydrophobicity. The specific surface area and pore structure of the samples were analyzed using a specific surface area and porosity tester (Gemini 2390, Micromeritics, Norcross, GA, USA).

### 2.5. Oil/Solvent Adsorption Experiments

In order to evaluate the oil adsorption performance of the synthesized aerogels, various oily liquids including paraffin oil, pump oil, kerosene, methylene chloride, vegetable oil, and dimethicone were selected as the adsorbate. In particular, a moderate mass of the hydrophobic COL/PVA/V_2_CT_x_ and COL/PVA composite aerogels was weighed using an analytical balance (0.01 mg, XDR226CDR, METTLER TOLEDO, Zurich, Switzerland), before being immersed in a container with 50 mL of oil or solvent. Then, the soaked aerogels were taken out and weighed quickly, when the equilibrium between the aerogels and oily liquids was reached. It is worth noting that excess oil/solvent on the surface of the aerogels must be cleaned using filter paper. The oil absorption capacities of the aerogels were calculated according to Equation (2) [36], as follows:

(2) $$C_{a}\left(g/g\right)=\frac{M-M_{0}}{M_{0}}\left(g/g\right)$$

where $C_{a}$ is the adsorption capacity of the aerogels for oil/solvent; *M* and *M*_0_ are the mass of the aerogels before and after adsorbing oil/solvent, respectively. Importantly, all the data were measured in triplicate and the average values were regarded as the final results in order to reduce the error of reproducibility.

## 3. Results and Discussion

### 3.1. Characterization of Prepared Composite Aerogels

**Figure 1:**
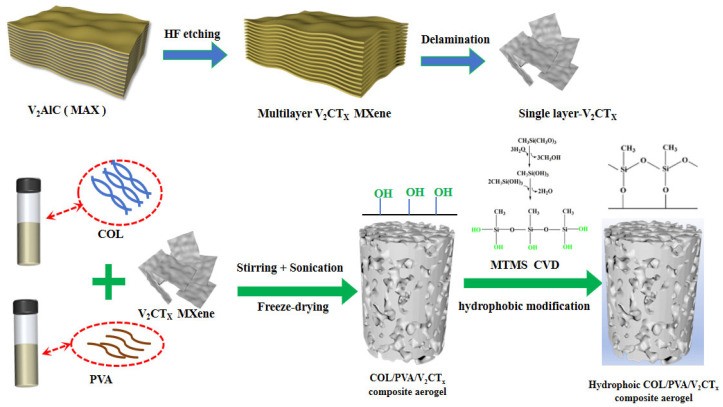
Flowchart of the preparation of hydrophobic COL/PVA/V_2_CT_x_ composite aerogels.

A schematic of the fabrication process of the hydrophobic COL/PVA/V_2_CT_x_ composite aerogels, including the processes of blending, stirring, sonication, directional freezing–drying, and modification via CVD, is illustrated in Figure 1. Firstly, the single V_2_CT_x_ MXene sheets were prepared by etching the ‘Al’ layer from V_2_AlC using HF, before delaminating using TBAOH. Secondly, the V_2_CT_x_ was dispersed in the COL and PVA mixed liquid with the help of oxygen-containing groups like hydroxyl and carboxyl that exist on the surface. The -CHO of GA can undergo a Schiff base reaction with -NH_2_ in collagen molecules, as well as a condensation reaction with the -OH in PVA, to form a network structure. The density of the aerogel rose with the increase in the amount of COL and PVA, which led to the network becoming denser. If their ratio deviated (potentially due to excessive collagen or excessive PVA), this caused an imbalance in the network structure, resulting in a mixture of large and small pores. Considering the influence of the mass concentration and ratio of the COL and PVA on the density and network structure of the composite aerogel, the dosage of both was determined to be 1 wt%. After the mixture containing COL, PVA, and V_2_CT_x_ MXene became homogeneous, the COL/PVA/V_2_CT_x_ composite aerogels were prepared using directional freezing–drying, which meant that the mixture was frozen from the bottom of the mold and ice crystals grew in one direction. When the mold containing the mixed liquid was completely frozen, the particles were expelled from the formed ice crystals due to phase separation and gathered around the growing ice crystals until a defined alignment structure was formed. Thirdly, the ice crystals were sublimated through lyophilization, and the porous composite aerogels were successfully prepared. In addition to hydrogen bonds, there is indeed an interfacial interaction between the inorganic phase of V_2_CT_x_ and the organic phase of COL and PVA. The V_2_CT_x_ can be embedded as a “physical cross-linking point” in the collagen/PVA network, which hinders the sliding of molecular chains and enhances mechanical strength. As a nucleation site, it regulates the growth of ice crystals (during freezing–drying) and induces the formation of a more uniform pore structure. Finally, the COL/PVA/V_2_CT_x_ composite aerogels were modified by means of chemical vapor deposition (CVD) with MTMS. The methoxy groups of MTMS reacted with water to form silanol (Si-OH), before undergoing condensation reactions with the hydroxyl groups (-OH) on the surface of the aerogels in order to take the shape of a stable silicon–oxygen bond (Si-O-Si) network [37]. Thus, the hydrophobic COL/PVA/V_2_CT_x_ composite aerogels were obtained.

**Figure 2:**
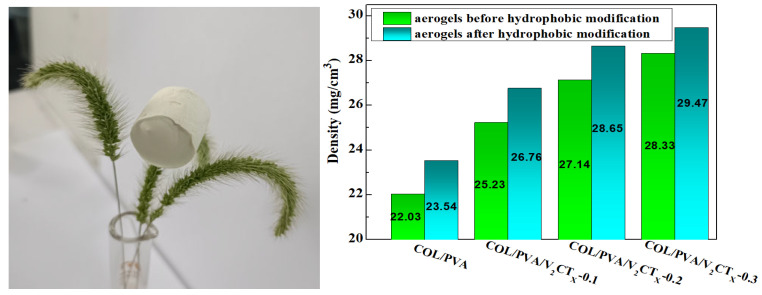
Density of the hydrophobic composite aerogels.

As shown in Figure 2, the prepared aerogels appeared to be light weight, as they could be placed on dog tail grass without obvious deformation. Moreover, the density of the hydrophobic COL/PVA/V_2_CT_x_ composite aerogel gradually increased, along with the mass ratio of V_2_CT_x_, because the total mass added remained unchanged. However, if too much V_2_CT_x_ (more than 0.3 wt%) was added, it was difficult for the mixture to become homogeneous and the V_2_CT_x_ MXene was easily deposited at the bottom during the freezing process due to aggregation. Meanwhile, the strength of the aerogel was enhanced with increasing density, which is essential so that the aerogels remain dimensionally stable after adsorbing oils. In addition, the strength of the prepared aerogel was enhanced with increasing density, which is important for ensuring dimensional stability after adsorbing oils [29]. Thus, MTMS-modified COL/PVA/V_2_CT_x_-0.2 seemed to be a good choice as an oil adsorbent.

**Figure 3:**
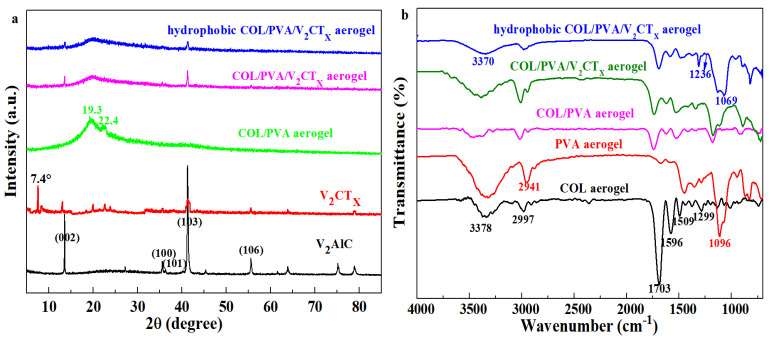
XRD patterns of V_2_AlC, V_2_CT_x_, and prepared aerogels (**a**). FTIR spectroscopy of COL, PVA, COL/PVA, COL/PVA/V_2_CT_x_, and hydrophobic COL/PVA/V_2_CT_x_ aerogels (**b**).

Figure 3a shows that the intensity of diffraction peaks at 13.3°, 35.5°, 36.3°, 41.2°, and 55.5° related to the crystal planes (002), (100), (101), (103), and (106) of V_2_AlC weakened or disappeared after etching and intercalation. The characteristic peak of 7.4° referred to the (002) plane of V_2_CT_x_ appears, which indicates the successful preparation of V_2_CT_x_ [38]. The variation in the peaks was caused by the reconstruction of crystal structure and the change in the parameters of crystal plane spacing. Meanwhile, the peak at 19.3°, which was appointed as a crystal plane (101) of PVA, and the diffraction peak at 22.4° related to numerous structural layers within collagen; both are observed in the XRD spectrum of the COL/PVA composite aerogel. Compared with the COL/PVA composite aerogel, the peaks relating to the (002) and (103) planes of V_2_CT_x_ are discovered in the patterns of the COL/PVA/V_2_CT_x_ composite aerogel_,_ which demonstrates that V_2_CT_x_ has been successfully introduced into the composite aerogel. Only the two strongest peaks of V_2_CT_x_ were shown, while the remaining peaks were not displayed in the XRD pattern, which might be due to V_2_CT_x_ being wrapped by collagen and polyvinyl alcohol, leading to the weaker peaks being difficult to detect. Meanwhile, there is no obvious difference between the curves of the COL/PVA/V_2_CT_x_ and hydrophobic COL/PVA/V_2_CT_x_ composite aerogels; the phenomenon shows that the CVD treatment does not damage the crystal structure of the products. Figure 3b reveals that the N-H stretching vibration (amide A) and -OH peaks at 3378 cm^−1^, the C=O stretching vibration peak of the amide I band at 1703 cm^−1^, the N-H bending vibration (amide II) peak at 1596 cm^−1^, and the C-N stretching vibration peak at 1299 cm^−1^ are characteristic peaks of the COL aerogel [14,39]. Additionally, the O-H stretching vibration peak at 3338 cm^−1^, the C-H stretching vibration peak of CH_2_ at 2941 cm^−1^, and the C-O stretching vibration peak of the alcoholic hydroxyl group at 1096 cm^−1^ are all typical peaks of the PVA aerogel. Meanwhile, all the representative peaks of COL and PVA are found in the spectra of the COL/PVA composite aerogel, confirming the preparation of aerogels by combing COL and PVA. In addition, no obvious variation between the COL/PVA and COL/PVA/V_2_CT_x_ composite aerogels is discovered, which shows that a new chemical bond is not formed in the synthesis of the COL/PVA/V_2_CT_x_ composite aerogel. After hydrophobic modification by MTMS, an antisymmetric stretching vibration peak of Si-O-Si at 1069 cm^−1^ and a symmetrical bending vibration peak of Si-C at 1236 cm^−1^ both appear in the pattern of the hydrophobic COL/PVA/V_2_CT_x_ composite aerogel [37]. In the meantime, the structure of the COL/PVA/V_2_CT_x_ composite aerogel is completely retained, with the peaks of COL and PVA still existing, determining that the structure of the aerogels is not affected by hydrophobic modification via CVD. Therefore, the hydrophobic COL/PVA/V_2_CT_x_ composite aerogel was synthesized by directional freezing–drying and chemical vapor deposition.

**Figure 4:**
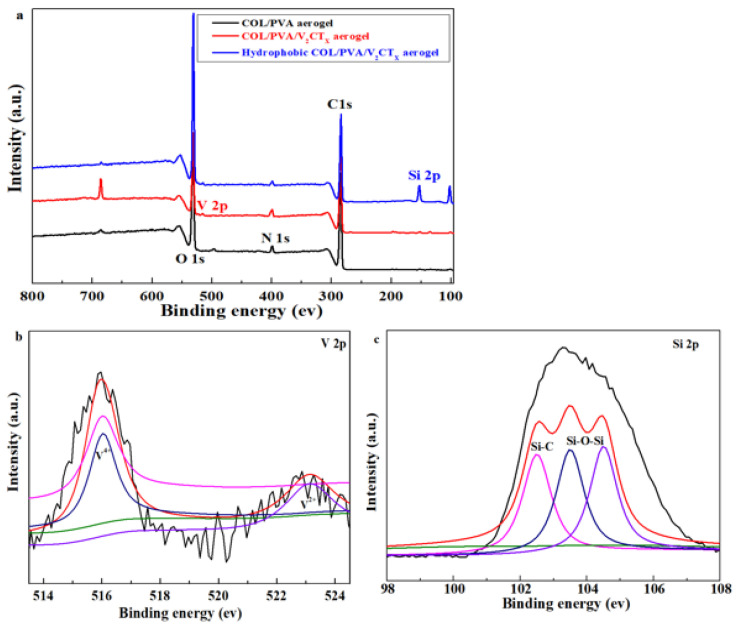
XPS spectra of the prepared aerogels (**a**); high-resolution XPS spectra of V 2p (**b**) and Si 2p (**c**) for the hydrophobic COL/PVA/V_2_CT_x_ composite aerogel.

As shown in Figure 4a, the peaks of C 1s at 285 eV, O 1s at 533 eV, and N 1s at 400 eV are all distinctly found in the XPS spectra of the COL/PVA, COL/PVA/V_2_CT_x_, and MTMS-modified COL/PVA/V_2_CT_x_ hybrid aerogels, which are the main peaks of collagen and polyvinyl alcohol [40]. Meanwhile, the peak at 516.1 eV, appointed to V 2p, can be seen for the COL/PVA/V_2_CT_x_ composite aerogel, which further confirms that the V_2_CT_x_ has been smoothly combined into the aerogel. In addition, the peak of V 2p and the peak at 102 eV—regarded as Si 2p—also appear in the hydrophobic COL/PVA/V_2_CT_x_ composite aerogel, which illustrates successful hydrophobic modification via VCD. The atomic concentrations of C 1s, O 1s, N 1s, and V 2p in the COL/PVA aerogel are 75.09%, 22.26%, 2.65%, and 0%, respectively, which turn into 77.22%, 19.03%, 3.55%, and 0.21% in the COL/PVA/V_2_CT_x_ aerogel. The change in atomic concentration is caused by the addition of V_2_CT_x_ with C, O, V, and other atoms. In particular, the Si 2p atomic concentration is shifted from 0% to 2.74% in the COL/PVA/V_2_CT_x_ composite aerogel before and after hydrophobic modification, which further reveals that the aerogel has been endowed with hydrophobicity. Figure 4b displays that the V 2p peaks at 516 eV and 523 eV are ascribed to V^4+^ and V^2+^. The peaks of Si 2p at 102.5 eV, 103.5 eV, and 104.5 eV are attributed to Si-CH, Si-CH_3_, and Si-O-Si, respectively, in Figure 4c, which indicates that the hydrophobic networks of Si-O-Si are formed in the aerogels, in accordance with the results of FTIR [35].

**Figure 5:**
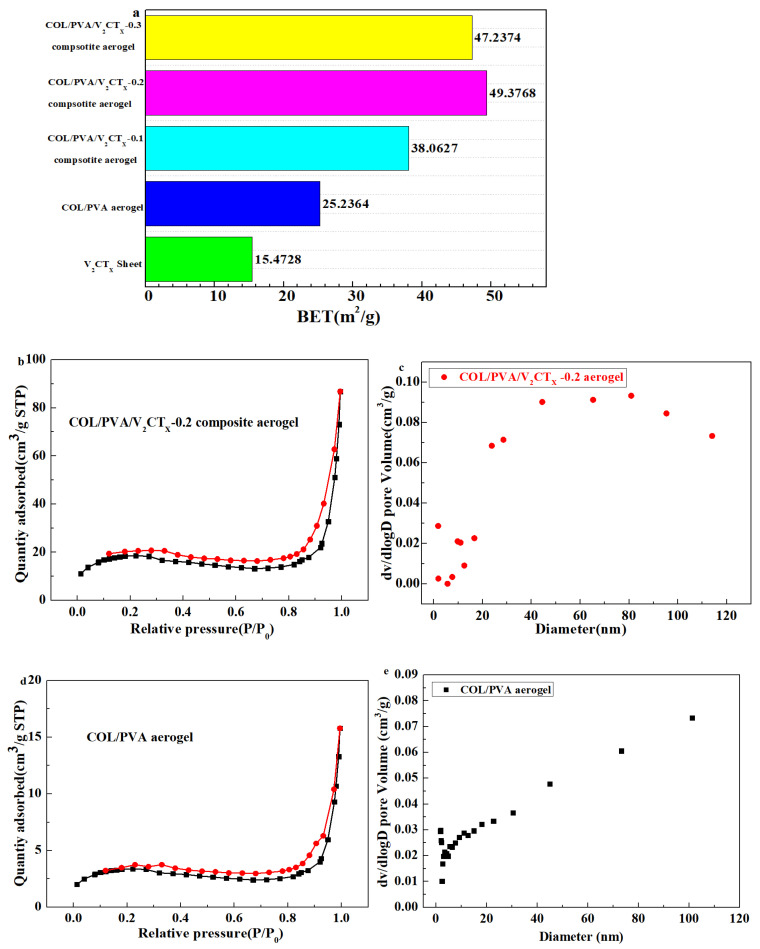
The specific surface area of prepared aerogels (**a**); N_2_ adsorption–desorption isotherms and pore size distribution of the hydrophobic COL/PVA (**b**,**c**) and COL/PVA/V_2_CT_x_-0.2 composite aerogels (**d**,**e**).

The specific surface area of the final aerogels was determined according to the Brunauer–Emmett–Teller (BET) method, while the pore size distribution was analyzed according to the Barrett–Joyner–Halenda (BJH) model from the N_2_ physical adsorption and desorption isotherms. Figure 5a shows that the specific area of the hydrophobic composite aerogels increases because of the addition of V_2_CT_x_. V_2_CT_x_ was evenly distributed on the surface and the layers of COL and PVA, which was conducive to constructing the three-dimensional porous structure and increasing the surface area of the composite aerogel. Meanwhile, the oxygen-containing groups on its surface formed intermolecular hydrogen bonds with the oxygen-containing groups of the collagen, which enhanced the stability of the porous structure and further prevented the collapse of the pore structure during the drying process, which was beneficial for the increase in the specific surface area. It should be noted that too much V_2_CT_X_ may clog the pores of the aerogel and could lead to a decrease in the specific surface area. From Figure 5b,d, the N_2_ adsorption and desorption isotherms of the aerogel formed an obvious hysteresis loop, which indicated that the aerogel formed a porous structure mainly containing mesopores. By comparing the pore size distribution of the aerogels in Figure 5c,e, it was found that the proportion of macropores in the COL/PVA/V_2_CT_x_—0.2 composite aerogel increased, which was helpful for adsorbing oils.

**Figure 6:**
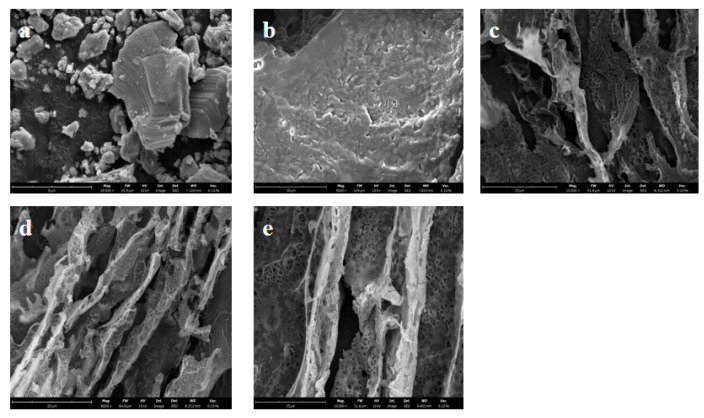
SEM images of V_2_AlC (**a**), V_2_CT_x_ MXene (**b**), the COL/PVA aerogel (**c**), the COL/PVA/V_2_CT_x_ composite aerogel (**d**), and the hydrophobic COL/PVA/V_2_CT_x_ hybrid aerogel (**e**).

The morphology of V_2_AlC MAX shows a tight and blocky structure in Figure 6a. The Al layer was removed by etching with HF, while delamination using TBAOH resulted in the V_2_CT_x_ MXene exhibiting a sheet structure, as shown in Figure 6b. As can be seen from the SEM photos in Figure 6c,d, the composite aerogel prepared by means of directional freezing–drying is porous and contains many holes that are created by the formation and growth of ice crystals during directional freezing. Meanwhile, large numbers of hole channels are formed in relation to the structure of COL and the treatment of directional freezing–drying. Due to the assistance of vanadium carbide in the formation of pores, the directional arrangement of the COL/PVA/V_2_CT_x_ composite aerogel is more obvious. After hydrophobic modification with MTMS via CVD, the pore structure of the COL/PVA/V_2_CT_x_ composite aerogel does not undergo obvious change, which implies that the porous 3D network structure with many hole channels remains and is helpful for oil and solvent adsorption.

**Figure 7:**
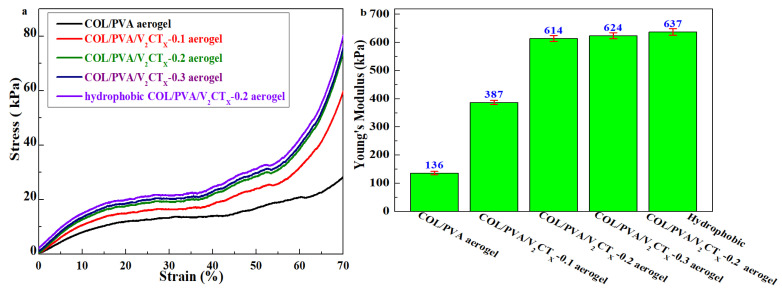
Typical stress–strain vertical axial compression curves (**a**) and Young’s modulus (**b**) of the aerogels.

The mechanical properties of aerogels are essential for their practical application; this was measured by vertical axial compression on aerogels in a cylindrical shape with a size of 18 mm in diameter and 15 mm in height. As shown in Figure 7a, three typical regions including a linear region, as well as steady and rapid growth regions, appear in the compression stress–strain curves of the prepared aerogels [29]. At low strains, the stress increases almost linearly with strain; this was identified as the first linear clastic deformation region. During compression, the stress increases slowly and steadily, entering the second plastic-yielding region. Finally, a sharp increase in stress is discovered at high strains, which is assigned as the third region. Figure 7a also reveals that the stress value of the COL/PVA/V_2_CT_x_ composite aerogel is higher than that of the COL/PVA aerogel at the same compressive deformation. In particular, the final stresses of the COL/PVA, COL/PVA/V_2_CT_x_—0.1, COL/PVA/V_2_CT_x_—0.2, and COL/PVA/V_2_CT_x_—0.3 composite aerogels at 70% strain are 28.5 kPa, 59.5 kPa, 71.5 kPa, and 73.5 kPa, respectively. As concerns the 2D V_2_CT_x_, the sheets play key roles in reinforcing material, and the internal structure constructed by the interaction between COL/PVA and V_2_CT_x_ MXene better withstands the negative impact during the compression of the aerogel. In addition, the stress of COL/PVA/V_2_CT_x_—0.2 slightly increased after hydrophobic modification with 76.5 kPa at 70% strain, which is caused by new chemical bonds such as the Si-O-Si and hydrogen bonds formed between MTMS and the aerogel. Meanwhile, Young’s modulus of the hydrophobic COL/PVA/V_2_CT_x_—0.2 composite aerogel is 637 kPa, which is nearly five times more than that of the COL/PVA aerogel (136 kPa), as shown in Figure 7b. The improvement in mechanical performance results from the addition of V_2_CT_x_ sheets, as well as the new chemical and hydrogen bonds formed during hydrophobic modification being consistent with XPS and FTIR characterization. Notably, the mechanically tested values of the hydrophobic COL/PVA/V_2_CT_x_ composite aerogel exceeded the level of some aerogel materials, as shown in Table 1; this is attributed to the vertical axial pore structure that is created by directional freezing–drying, the superiority of the raw materials of COL and PVA, the addition of V_2_CT_x_, and modification by MTMS, which leads to new chemical bonds and hydrogen bonds emerging. The excellent mechanical properties promote the practical application of aerogels in relation to oil adsorption, which is helpful for maintaining the shape, structure, and reusability in adsorption process.

**Figure 8:**
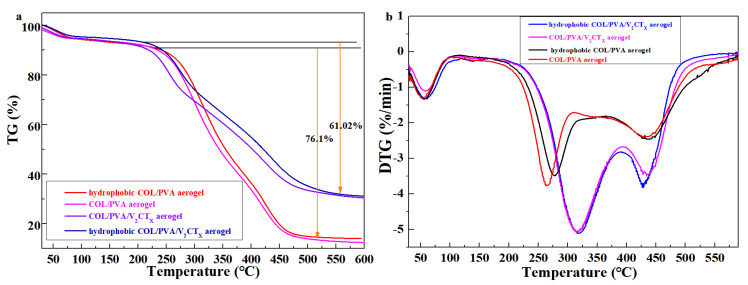
TGA curves (**a**) and DTG curves (**b**) of the prepared aerogels.

Figure 8a shows the TGA curves of the aerogels, which are used to evaluate the thermal stability of the materials. In Figure 7a, the residual weight percentage of the COL/PVA composite aerogel is 13.17%, while that of the COL/PVA/V_2_CT_x_ aerogel is 31.35%, which results from the addition of V_2_CT_x_ at a higher decomposition temperature. Meanwhile, the weight loss of the COL/PVA/V_2_CT_x_ aerogel decreases 61.02% from 208 °C to 550 °C, which is less than that (76.1%) of the COL/PVA aerogel from 205 °C to 520 °C. After hydrophobic modification using MTMS, the residual weight percentage of the aerogels increases and the weight loss reduces, which is caused by the rigid polysiloxane coating slowing down the thermal decomposition of the modified aerogels. Moreover, the DTG curves in Figure 8b indicate that the COL/PVA/V_2_CT_x_ aerogel thermally decomposed fastest at 320 °C, which is higher than the temperature (260 °C) of the COL/PVA aerogel’s decomposition. The fastest thermally decomposed temperature of the aerogels after hydrophobic treatment also improves due to the reaction between the aerogels and MTMS. Therefore, the hydrophobic COL/PVA/V_2_CT_x_ composite aerogel with a better thermal stability is regarded as a new oil adsorbent that can be applied in worse environments, such as those with high temperatures.

### 3.2. Oil Adsorptive Performance of Aerogels

**Figure 9:**
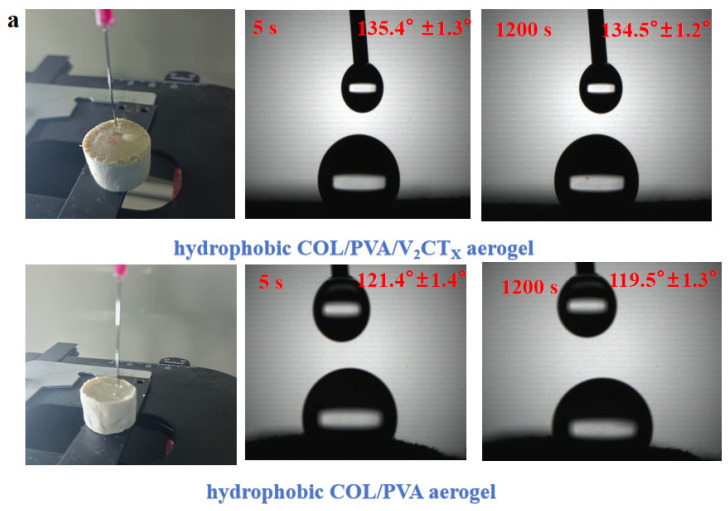

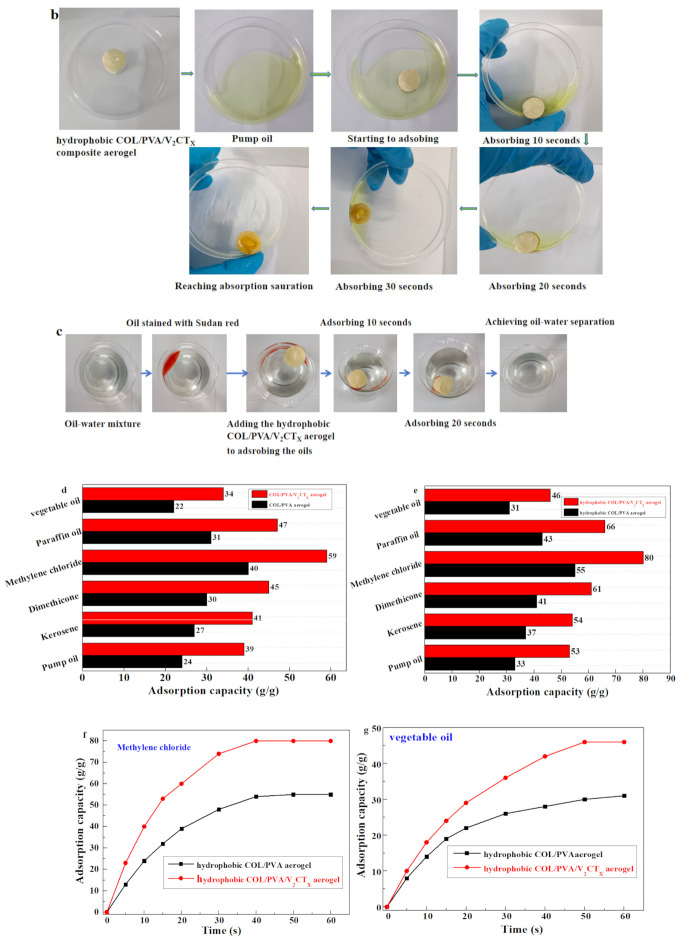

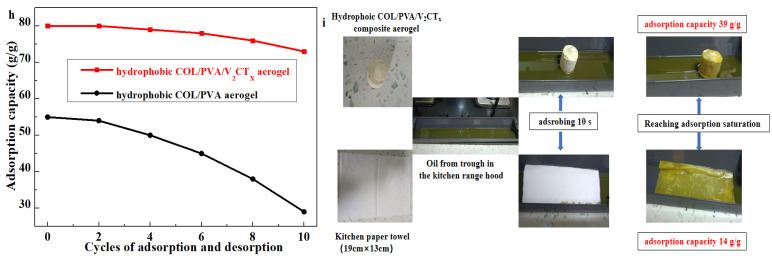
The water contact angle of hydrophobic aerogels taken at 5 s and 1200 s (**a**). The process of the hydrophobic COL/PVA/V_2_CT_x_ composite aerogel adsorbing pump oil (**b**). The selective removal of dimethyl silicone oil from the water surface (**c**). The adsorption capacities of aerogels (**d**) and hydrophobic aerogels (**e**) for different kinds of oily liquids. The adsorption capacity of aerogels for methylene chloride (**f**) and vegetable oil (**g**) in relation to adsorbing time. The adsorption capacity of aerogels on cycles of adsorption and desorption (**h**). The practical application of composite aerogel in adsorbing residual kitchen oil (**i**).

The modified COL/PVA/V_2_CT_x_—0.2 composite aerogel was selected to adsorb oils; it has the advantages of being ultra lightweight, has a large specific area and porous structure, is hydrophobic, has compression properties, and is thermally stable. After silylation by MTMS, the hydrophobicity was introduced into the COL/PVA/V_2_CT_x_ composite aerogel and water drops fell on the surface, maintaining its round shape with a contact angle of 135.4° for 5 s and 134.5° for 1200 s (Figure 9a). The water contact angle of the hydrophobic COL/PVA composite aerogel is 121.4° for 5 s and 119.5° for 1200 s, which is less than that of the hydrophobic COL/PVA/V_2_CT_x_ composite aerogel, which is attributed to the hydrophobicity of V_2_CT_x_. Figure 9b describes the absorption process of the hydrophobic COL/PVA/V_2_CT_x_ composite aerogel for pump oil. The aerogel still maintains its original size after reaching adsorption saturation in 30 s, benefitting from its mechanical properties. Efficient oil/water selectivity is also an important property for oil adsorbents, in particular for removing oil spills in aqueous environments. As displayed in Figure 9c, the MTMS-modified aerogel was dipped into the mixed liquid composed of dimethyl silicone oil and water; the oil was selectively adsorbed by the aerogel to finish the oil–water separation in 20 s. It should be noted that the aerogel, which was lightweight, porous, and hydrophobic, continued to float on the surface of the water without releasing oil after adsorbing oils, which is beneficial for rapidly and efficiently removing oils in practical applications relating to oil spills.

Moreover, the prepared COL/PVA/V_2_CT_x_ and COL/PVA composite aerogels before and after hydrophobic modification were applied to adsorb various oils and solvents such as pump oil, vegetable oil, paraffin oil, kerosene, dimethicone, and methylene chloride; the absorption capacity is shown in Figure 9d,e. The results show that the adsorption capacities of the aerogels for oils were promoted after being modified by MTMS; this can be attributed to the introduction of the hydrophobic effect. The adsorption capacities of the hydrophobic COL/PVA/V_2_CT_x_ aerogel ranged from 46 to 80 g/g, which is more than that (31–55 g/g) of COL/PVA, toward different types of oily liquids; this can be explained by the uniform alignment of numerous pores providing more space for oils, a bigger specific surface area offering more active sites for adsorbing oils, and a larger water contact angle with a stronger hydrophobic effect. In addition, the adsorption capacity is also affected by the density, viscosity, and surface tension of the adsorbed oil. Oils were adsorbed into the aerogel and were primarily stored in the macropores with the help of capillary forces and hydrophobic interactions.

Among the adsorbed oils, the viscosity of vegetable oil is highest (44.5 mPa·s), while the viscosity of dichloromethane is lowest (0.43 mPa·s). Figure 9f,g reveal the relationship between the adsorption capacity of aerogels and adsorbing time. Compared with the hydrophobic COL/PVA composite aerogel, it took less time for COL/PVA/V_2_CT_x_ to reach adsorption saturation, which benefited from the porous structure with more channels, larger specific surface area, and greater hydrophobic effect. In particular, 40 and 50 s were needed for the hydrophobic COL/PVA/V_2_CT_x_ aerogel to completely adsorb methylene chloride and vegetable oil, which is less than the time needed by the hydrophobic COL/PVA composite aerogel. Meanwhile, the adsorption saturation of methylene chloride was more quickly reached in comparison to vegetable oil, which resulted from the viscosity of methylene chloride being less than that of vegetable oil, leading to the oil’s molecules moving more quickly. Therefore, the adsorption saturation for vegetable oil with a higher viscosity was achieved in a longer time. As shown in Figure 9f,g, the adsorption rate of modified aerogels for various oils was fast at the start but gradually reduced with time until the absorption process was completed. The variation in adsorption rate is explained by the available active sites that exist in the aerogels gradually decreasing until they are completely occupied by the adsorbed oil. The recyclability of adsorbents is a very important indicator in practical applications.

The reusability of adsorbents that are affected by mechanical properties is critical for practical applications. The adsorption capacity of modified aerogels for methylene chloride was tested after adsorption–desorption (squeezing and distillation), as shown in Figure 9h. The adsorption capacity of the hydrophobic COL/PVA/V_2_CT_x_ composite aerogel is maintained at about 90% of the initial value after 10 cycles, while the adsorption capacity of the hydrophobic COL/PVA aerogel decreases to 50%, which confirms that its reusability is better. The results of the fabricated aerogel and Vinda kitchen paper adsorbing the oil in the oil pan of a household kitchen range hood are shown in Figure 9i. The adsorption capacity of the COL/PVA/V_2_CT_x_ composite aerogel reached 39 g/g, which was about three times the adsorbing quantity of the kitchen paper. Moreover, the MTMS-modified COL/PVA/V_2_CT_x_ aerogel had a higher adsorption capacity compared with other oil adsorbents that were reported in Table 2; this promises to be a novel and practical oil adsorbent as it is environmentally friendly and easy to fabricate.

### 3.3. Analysis of Oil Adsorption Kinetics and Mechanisms

**Figure 10:**
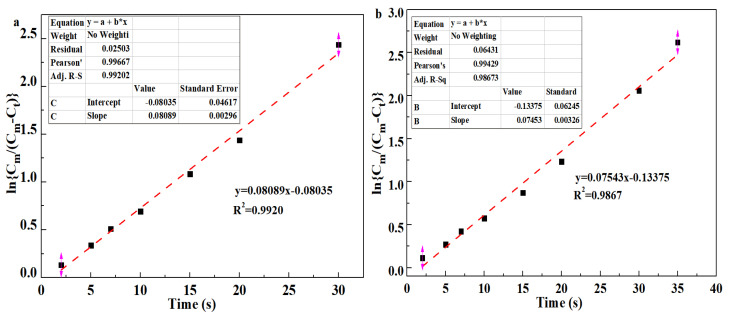

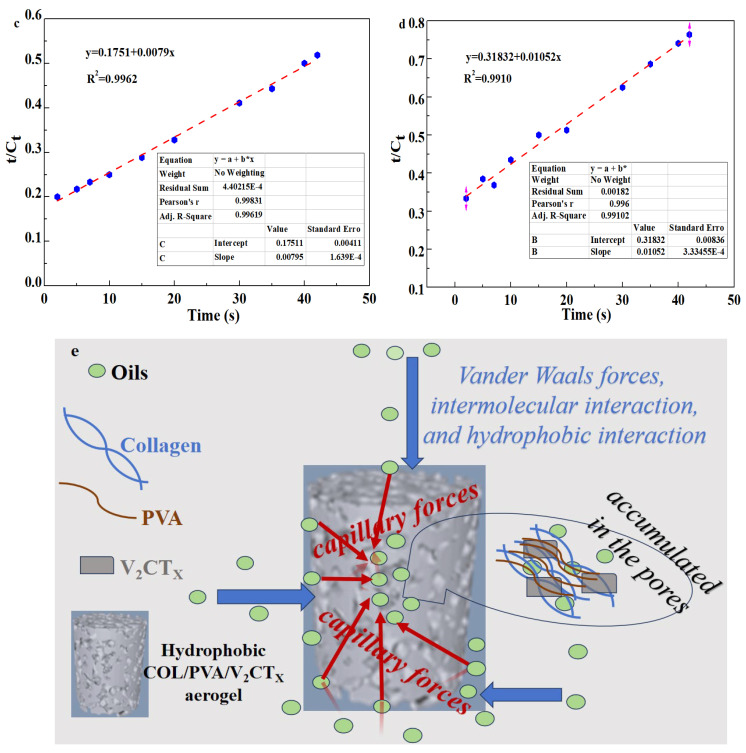
Pseudo first−order absorption linear fitting of (**a**) the COL/PVA/V_2_CT_x_ aerogel and (**b**) the COL/PVA aerogel for methylene chloride. Pseudo second−order absorption linear fitting of (**c**) the COL/PVA/V_2_CT_x_ aerogel and (**d**) the COL/PVA aerogel for methylene chloride. The adsorption mechanism of the COL/V_2_CT_x_ aerogel for oils (**e**).

Pseudo first-order and pseudo second-order models are commonly and widely applied among various kinetics models of oil adsorption; they can be obtained by fitting the experimental data, respectively, to Equations (3) and (4) [45], as follows:

(3) $$ln\frac{C_{m}}{C_{m}-C_{t}}=k_{1}t$$

(4) $$\frac{t}{C_{t}}=\frac{1}{k_{2}C_{m}^{2}}+\frac{t}{C_{m}}$$

where *C_m_* is the maximum oil adsorbance of aerogels, *C_t_* is the oil adsorbance of aerogels at time *t*, *k*_1_ is the adsorption rate constant of the pseudo first-order model obtained from the slope of the fitted line, *k*_2_ is the rate constant of the pseudo second-order model determined by the intercept of fitted line, and *t* is the adsorbing time. Figure 10a,b show the pseudo first-order model of the hydrophobic COL/PVA/V_2_CT_x_ and COL/PVA composite aerogels adsorbing methylene chloride, while Figure 10c,d reveal the pseudo second-order model of the aerogels. Significantly, the correlation coefficient R^2^ of the hydrophobic COL/PVA/V_2_CT_x_ and COL/PVA composite aerogels (0.9920 and 0.9867) fitted using the pseudo first-order model is lower than that (0.9962 and 0.9910) fitted using the pseudo second-order model, which demonstrates that the oil adsorption behavior can be more compatibly predicted using the pseudo second-order model containing physical and chemical adsorption. Moreover, the adsorption rate constant *k_1_* calculated by the slope of the hydrophobic COL/PVA/V_2_CT_x_ composite aerogel is 0.0809, which is higher than that (0.0754) of the COL/PVA aerogel. Meanwhile, the adsorption rate constant *k*_2_ is 0.00037, which is higher than that (0.00035) of the COL/PVA aerogel. These results indicate that the adsorption rate of the hydrophobic COL/PVA/V_2_CT_x_ aerogel is faster.

Figure 10e describes the adsorption process of the prepared aerogels in relation to various oils. Firstly, the oils were adsorbed onto the aerogel surface via the action of the Van der Waals forces, intermolecular interactions, and hydrophobic effects. Secondly, the oil flowed into the aerogel interior with the assistance of capillary forces. Lastly, the oil was stored in the pores of the aerogels [10]. The molecular structure of oily liquids such as oils and hydrocarbons is mainly composed of carbon and hydrogen chains, making them typical non-polar substances. According to the principle of interfacial energy minimization, the oil is more easily adsorbed by the hydrophobic surface by forming a stable interfacial bond [46]. The internal pores of the hydrophobic COL/PVA/V_2_CT_x_ composite aerogel generate capillary forces and porous channels. The larger specific surface area provides more active sites for adsorbing oils. Therefore, the oil is easily suctioned into the pore interior by capillary force through the channels with the help of intermolecular forces and hydrophobic effects. Hence, the hydrophobicity; specific surface area; pore structure, including pore size and distribution; and quantity of aerogels are all important for adsorption capacity. The hierarchical structure between the pores provides more adsorption channels for oils, the large specific surface area provides more active sites, and the multi-level porous structure offers more oil storage space. In addition, the viscosity and density of the oils also play key essential roles in relation to adsorption capacity.

## 4. Conclusions

The hydrophobic, light, and porous COL/PVA/V_2_CT_x_ composite aerogel was prepared by directional freezing–drying and modification using MTMS; it used the following raw materials: collagen (type Ⅰ), polyvinyl alcohol, and two-dimensional V_2_CT_x_ MXene. The structure, composition, and morphology of the aerogels were characterized using XRD, FTIR, XPS, and SEM measurements. The hydrophobic COL/PVA/V_2_CT_x_ composite aerogel is equipped with a high strength, with 80.5 kPa stress at 70% strain, and thermal stability, with a 61.02% weight loss from 208 °C to 550 °C. The adsorption capacity of the COL/PVA/V_2_CT_x_ composite aerogel with hydrophobicity for various oils is up to 80 times its own weight, which is higher than that of the COL/PVA aerogel, revealing excellent reusability and a faster adsorption rate. Moreover, the adsorption performance of the prepared aerogels is predicted using the pseudo second-order model with a high correlation coefficient R^2^. The oily liquid is adsorbed onto the surface of the aerogels with the help of Van der Waals forces, intermolecular interactions, and hydrophobic effects, and flows into the aerogel interior through the porous channel by capillary forces and hydrophobic interactions; it is gathered and stored in the pores of the aerogels. Therefore, the COL/PVA/V_2_CT_x_ composite aerogel with hydrophobicity shows great prospects in the field of oil adsorption.

## Figures and Tables

**Table 1:** Comparison of the compressive mechanical properties of different materials.

Materials	Stress at Strain	Modulus (kPa)	Ref.
WC-Col aerogel	10 kPa at 70%	4.29	[17]
GO/PVA/CNF aerogel	6.8 kPa at 50%	-	[29]
Graphene aerogels	21.5 kPa at 50%	-	[3]
Xyloglucan/cellulose nanocrystals aerogels	75 kPa at 70%	360	[13]
CCF/MXene aerogel	3 kPa at 14%	3.3	[40]
Hydrophobic COL/PVA/V_2_CT_x_—0.2 aerogel	80.5 kPa at 70%	637	This work

**Table 2:** Comparison of the adsorption capacities of various materials.

Adsorbents	Oils	Adsorption Capacity (g/g)	Ref.
Chitosan–silica aerogel	Pentane, hexane, acetone, etc.	13–30	[41]
MTMS-coated cellulose aerogel	Motor oil and cooking oil	18–20	[42]
TMCS/rGO/CNF aerogel	Soybean oil, corn oil, and vacuum oil	33–39	[32]
Cellulose aerogel	Motor oil and machine oil	42–46	[2]
Polydopamine/MXene Nanosheet/Polyaniline/Polydimethylsiloxane foams	Soybean oil, toluene, and chloroform	13–61	[43]
S-Fe_3_O_4_/MXene/lignin@PU sponge	CCl_4_, PEG, edible oil, liquid paraffin, n-hexane, and DMF	29–70	[44]
TiVCT_X_ MXene/Graphene Nanosheet-Based Aerogels	Pump oil	42	[28]
Hydrophobic COL/PVA/V_2_CT_x_ aerogel	Pump oil, methylene chloride, vegetable oil, etc.	46–80	This work

## Data Availability

Any data and information used to support the research are present in the report.
